# Supplementary figures and images for: The human RNA polymerase I structure reveals an HMG-like docking domain specific to metazoans
Source: Life Sci Alliance. 2022 Sep 1;5(11):e202201568. doi: 10.26508/lsa.202201568 (PMC9438803; doi:10.26508/lsa.202201568)

RPA1

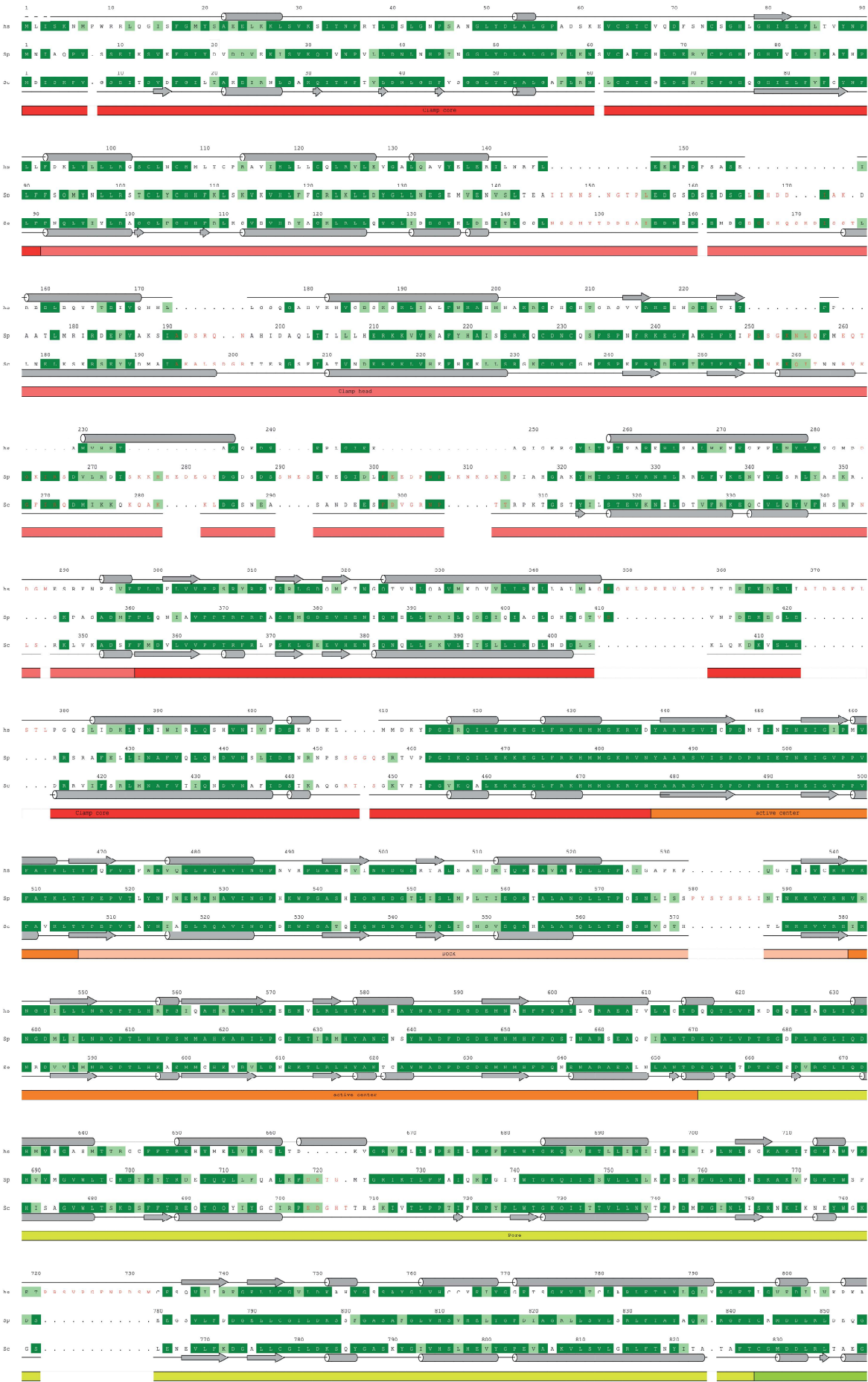

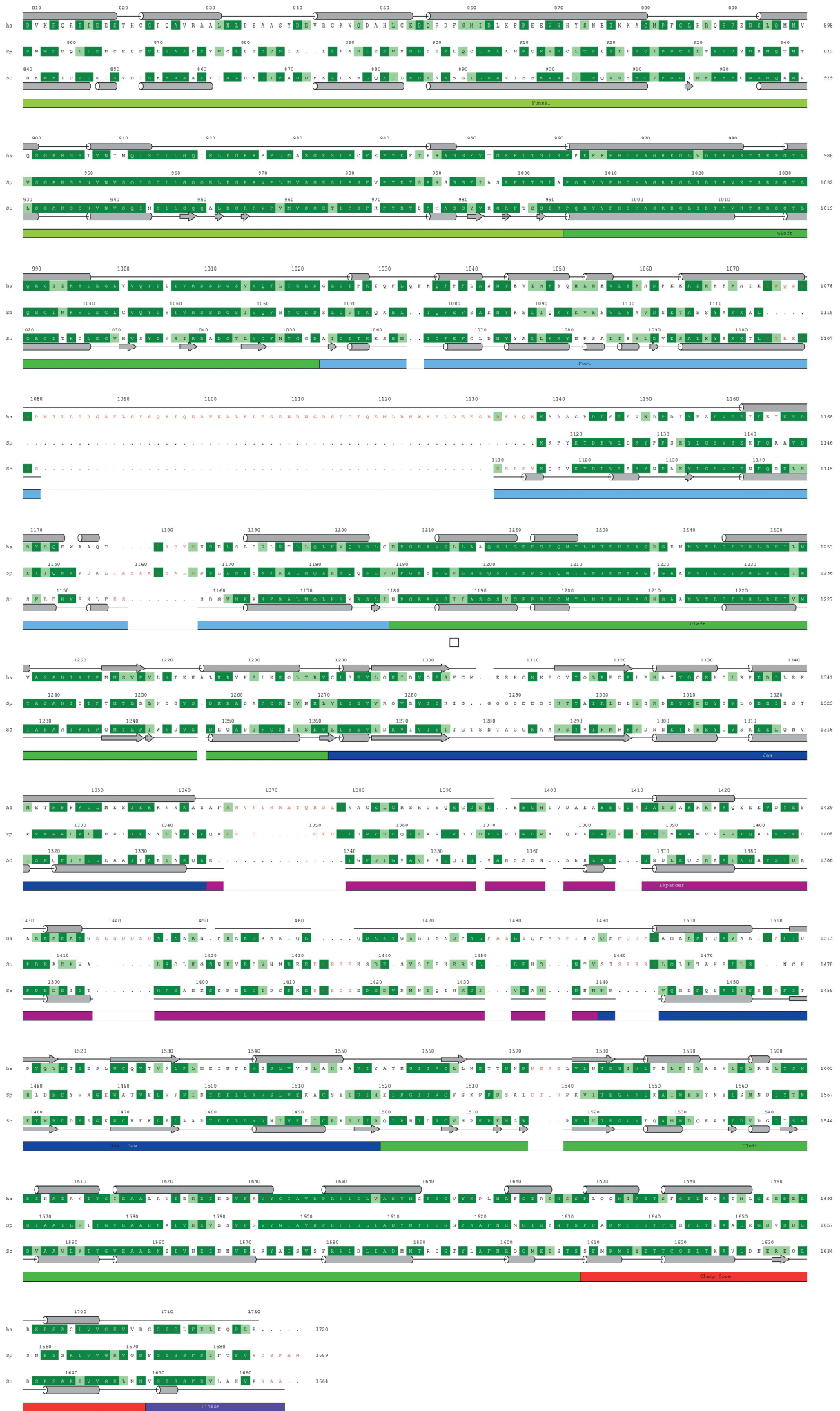

## RPA2

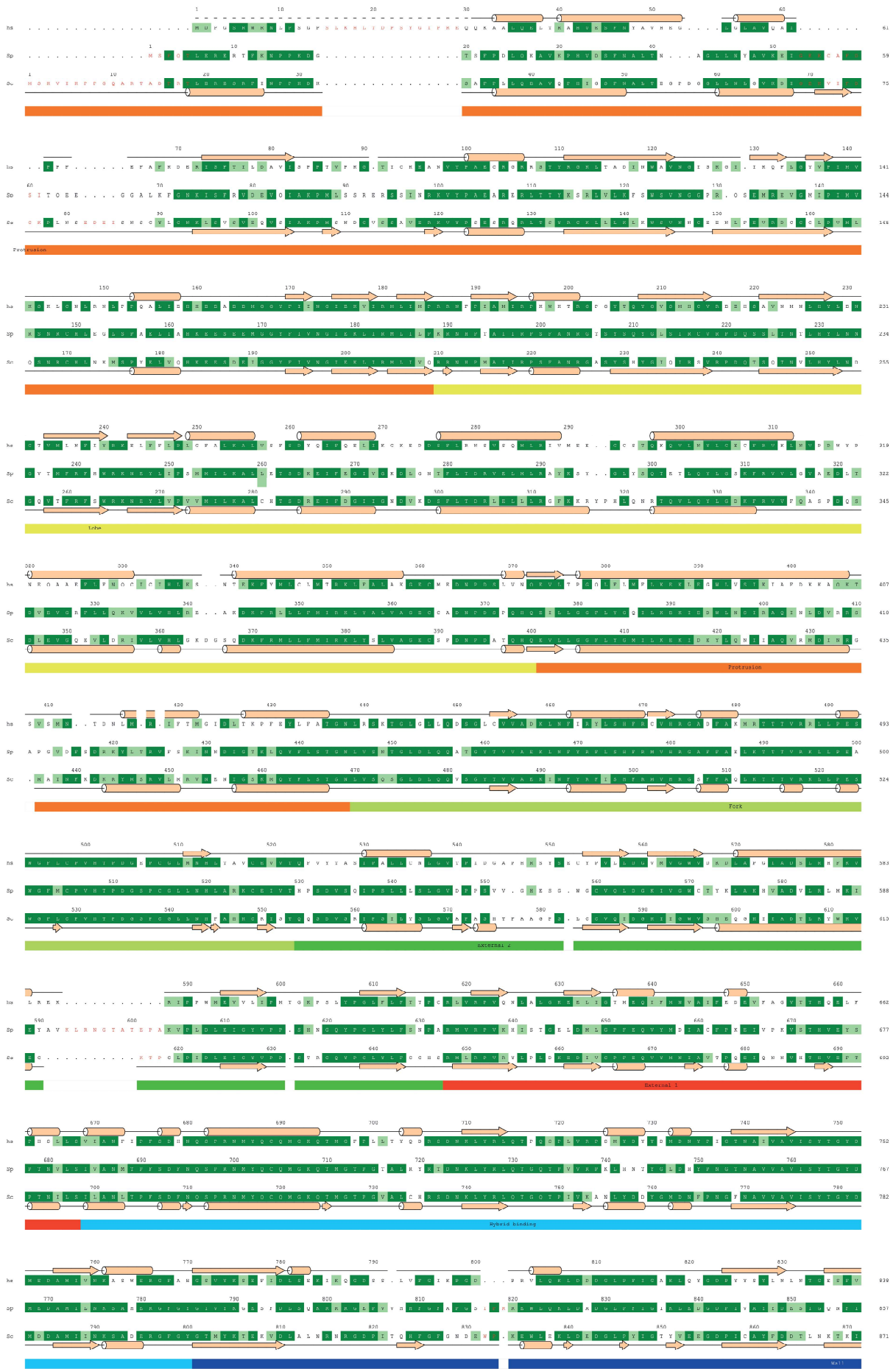

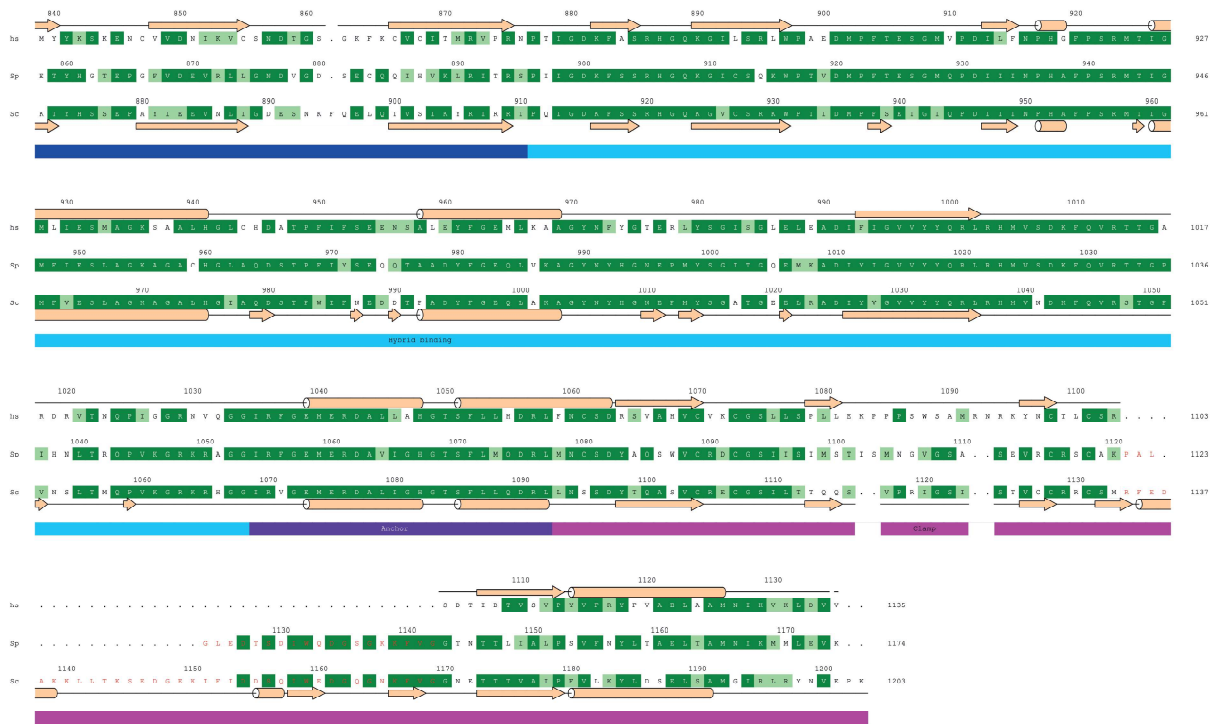

# RPAC1

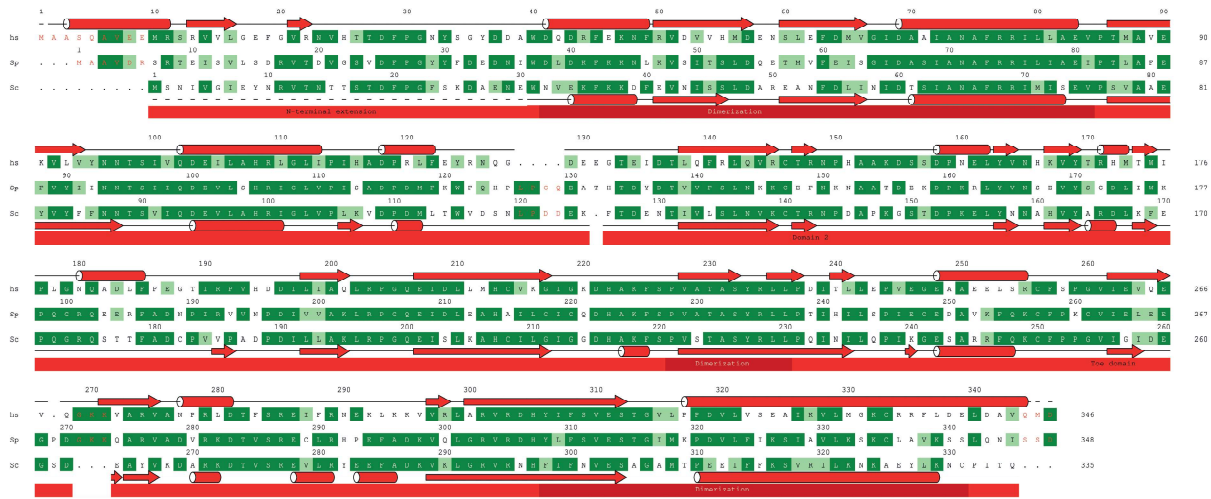

# RPAC2

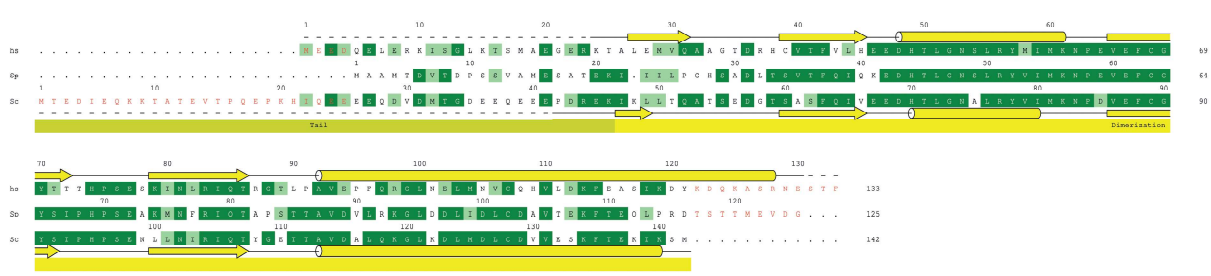

RPA49

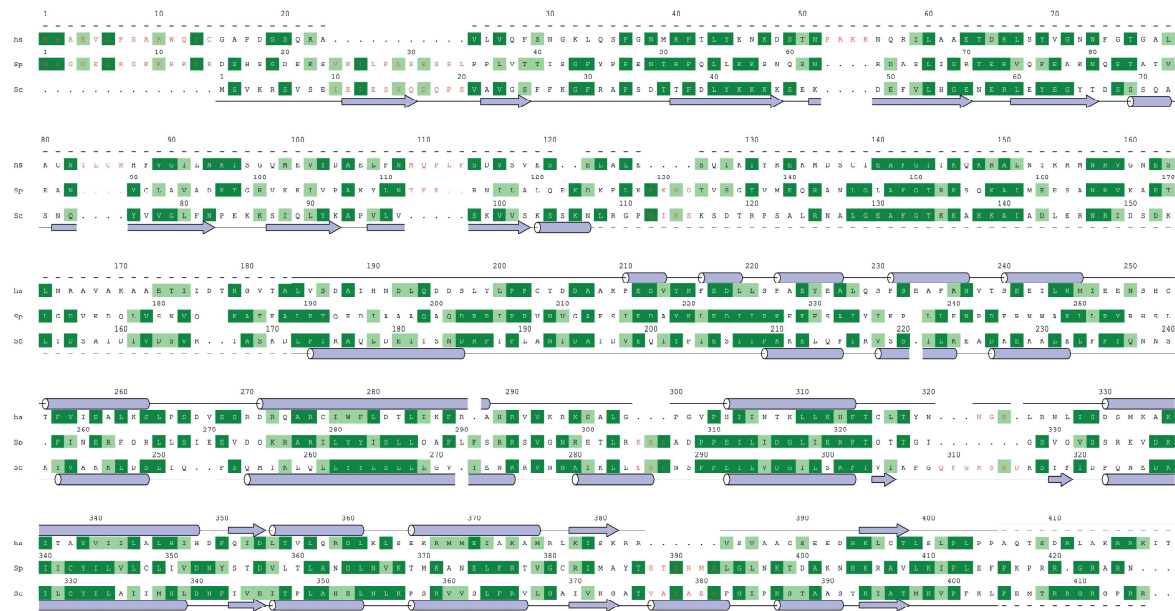

RPA34

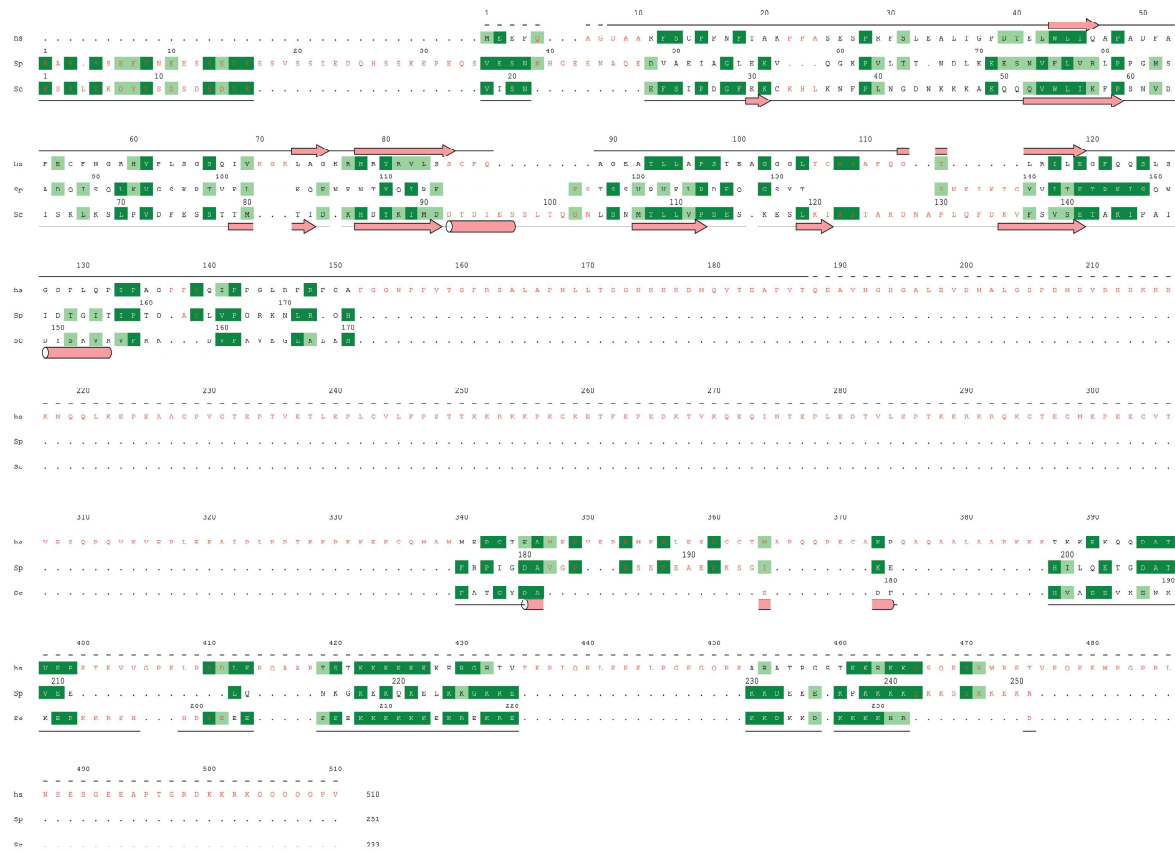

RPA12

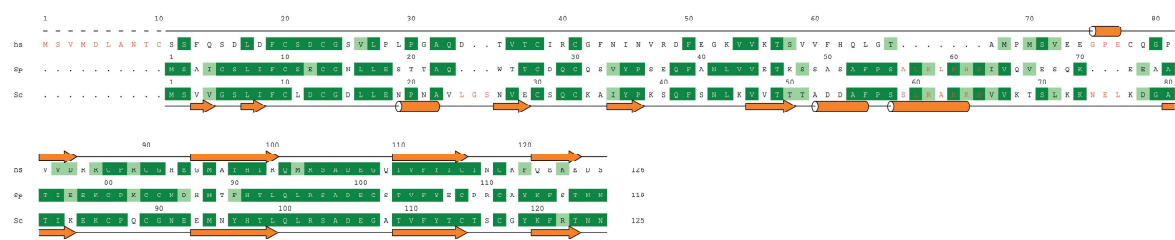

RPABC1

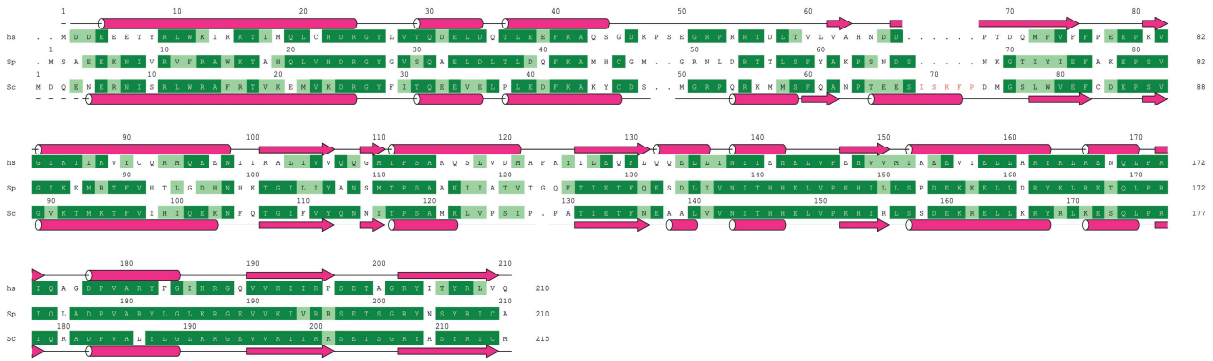

RPABC2

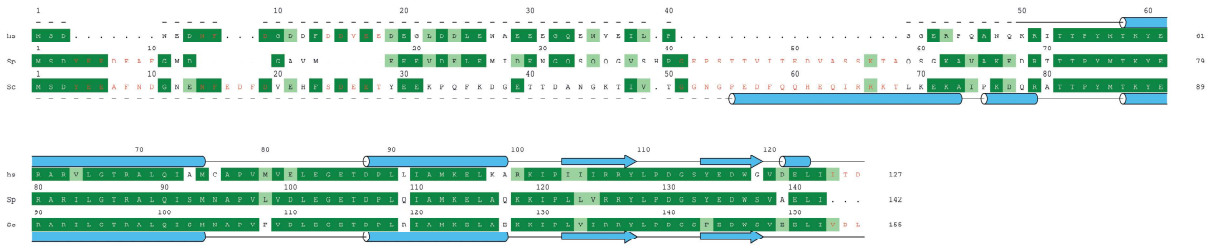

RPABC3

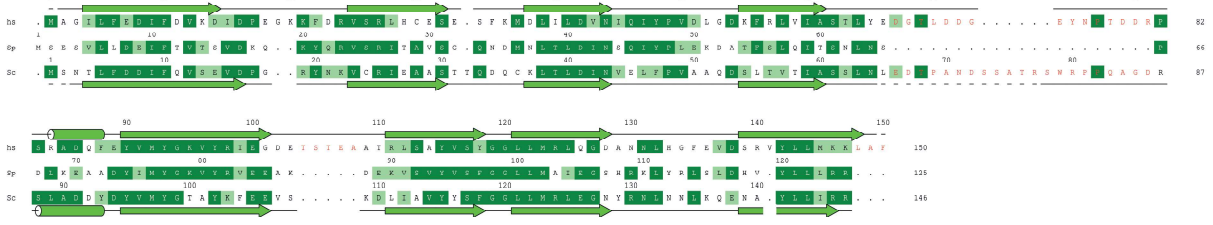

RPABC4

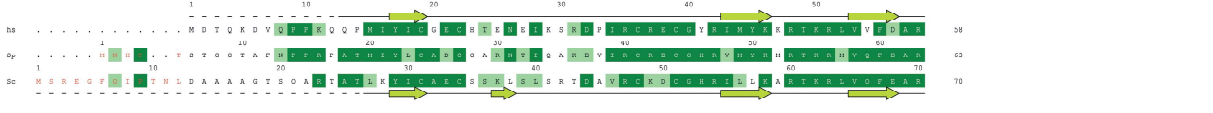

RPABC5

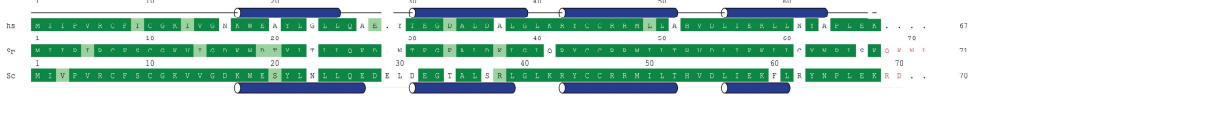

RPA43

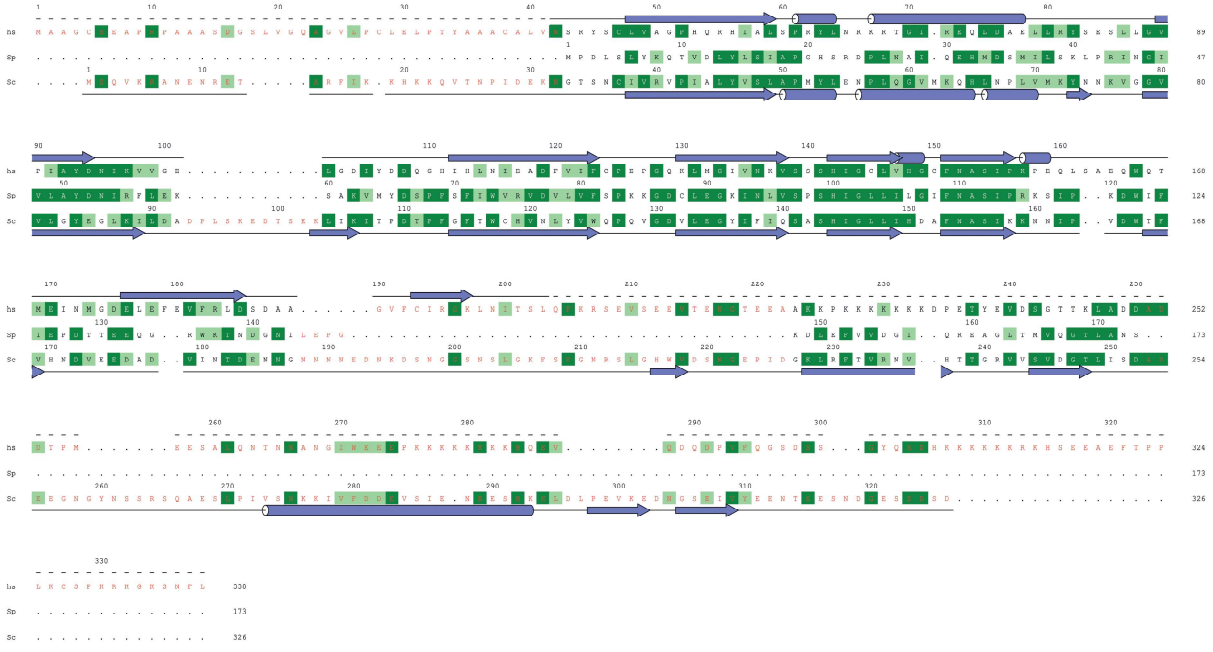

Supplement: Supplementary file 1 [file LSA-2022-01568_Supplemental_Data_1.pdf]
