# Supplementary material for: The human RNA polymerase I structure reveals an HMG-like docking domain specific to metazoans
Source: Life Sci Alliance. 2022 Sep 1;5(11):e202201568. doi: 10.26508/lsa.202201568 (PMC9438803; doi:10.26508/lsa.202201568)
Supplement: Supplementary file 2 [file LSA-2022-01568_TableS1.docx]

Supplementary Tables and their legends

**Table S1. Cryo-EM data collection and refinement statistics**

| PDB  EMDB | Human Pol I  8A43  15135 |
| --- | --- |
| **Data collection and processing** |  |
| Magnification | 50.000 |
| Voltage (kV) | 200 |
| Electron exposure (e^-^/Å^2^) | 40 |
| Defocus range (µm) | -1.2 - -2.7 |
| Pixel size (Å) | 0.968 (binned to 1.5085) |
| Symmetry imposed | C1 |
| Initial particles images (no.) | 145,554 |
| Final particle images (no.) | 108,012 |
| Map resolution (Å)  FSC threshold | 4.09  0.143 |
| **Refinement** |  |
| Initial model used (PDB code) | 5M3M |
| Model resolution (Å)  FSC threshold | 3.5-4.1 Å  0.143 |
| Model composition  Non-hydrogen atoms  Protein residues  Nucleotides  Ligands | 31,110  3,914  -  - |
| B factors (Å^2^)  Protein  Nucleotides  Ligand | 212.67  -  - |
| R.m.s. deviations  Bond lengths (Å)  Bond angles (°) | 0.008  1.005 |
| Validation  MolProbity score  Clashscore  Poor rotamers (%) | 2.54  23.93  0.41 |
| Ramachandran plot  Favored (%)  Allowed (%)  Disallowed (%) | 84.64  15.16  0.21 |
