# Supplementary material for: The human RNA polymerase I structure reveals an HMG-like docking domain specific to metazoans
Source: Life Sci Alliance. 2022 Sep 1;5(11):e202201568. doi: 10.26508/lsa.202201568 (PMC9438803; doi:10.26508/lsa.202201568)
Supplement: Supplementary file 3 [file LSA-2022-01568_TableS2.docx]

Supplementary Tables and their legends

**Table S2. Interactions within the *in situ* predicted complexes between human Top2a the RPA1 subunit of Pol I.** Listed are all inter-protein hydrogen bonds between the largest subunit of hPol I and human Topoisomerase 2a of the predicted complexes shown in Figure S8A.

| A |  | B |  | C |  | D |  |
| --- | --- | --- | --- | --- | --- | --- | --- |
| hPol I | hTop2a state I | hPol I | hTop2a state I | hPol I | hTop2a state II | hPol I | hTop2a state II |
| ARG814A | GLU1072A | ASN1081A | GLN1190A | SER1077A | TYR640A | LYS1074A | GLU55B |
| GLU818A | LYS1075A | LYS1095A | VAL1191A | LYS1095A | THR453A | GLU1130A | LYS233B |
| GLU818A | GLN1079A | LYS1101A | GLN585B | LYS1137A | ARG568A | SER1131A | ASP65B |
| GLU835A | ARG1053A | LYS1101A | GLU589B | GLU1179A | ASP659A | SER1131A | LYS233B |
| TYR872A | GLN1079A | LYS1104A | GLU597B | GLU1179A | ASP660A | ARG1133A | ASP234B |
| GLU875A | LYS1071A | GLN1172A | GLN1206A | LYS1180A | ASP659A | LYS1137A | GLU74B |
| ASN1081A | ASP671A | LYS1180A | GLU1209A | ARG1193A | GLU644A | GLN1172A | ASN294A |
| GLN1094A | GLU663A | LYS1272A | SER812A | GLU1521A | LYS265B | LYS1180A | ASP252A |
| LYS1095A | ASP660A | LYS1272A | ARG815A | GLU1521A | LYS265B | TYR1182A | ASP252A |
| LYS1095A | GLU663A | LYS1272A | GLU1177A |  |  | LYS1273A | ALA496B |
| GLU1098A | ASP660A | CYS1332A | GLU1150A |  |  | LYS1273A | SER497B |
| ALA1099A | ASP660A | ARG1334A | ASP1142A |  |  | LYS1276A | ALA496B |
| LYS1101A | ASP659A | THR1519A | LYS1141A |  |  | SER1280A | LYS656B |
| ARG1133A | PRO643A | GLU1520A | ARG1053A |  |  | GLN1328A | ASP660B |
| LYS1272A | HIS1005A | GLU1520A | ARG1148A |  |  | GLU1336A | LYS662B |
| LYS1273A | ASP1021A | GLU1521A | ARG1148A |  |  | ARG1501A | ASP1013B |
| LYS1273A | GLU1024A | GLU1521A | ASN1149A |  |  | TYR1517A | ASP1013B |
| LYS1276A | GLU1024A | LYS1571A | GLU1179A |  |  | GLU1520A | TRP860B |
| LYS1571A | LEU678A | LYS1574A | GLU1176A |  |  | GLU1520A | LYS1010B |
