# Supplementary material for: The human RNA polymerase I structure reveals an HMG-like docking domain specific to metazoans
Source: Life Sci Alliance. 2022 Sep 1;5(11):e202201568. doi: 10.26508/lsa.202201568 (PMC9438803; doi:10.26508/lsa.202201568)
Supplement: Supplementary file 4 [file LSA-2022-01568_TableS3.docx]

Supplementary Tables and their legends

**Table S3. DNA/**RNA oligonucleotides used in this **study**

| Name | Sequence |
| --- | --- |
| EC_a-template-DNA | 5’-CGAGGTCGAGCGTTGTCCTGGT-3’ |
| EC_a-bubble-template-DNA | 5’-CGAGGTCGAGCGTTGTCCTGGTGCTACGACGATCAG-3’ |
| EC_b-template-DNA | 5’-CGAGGTCGAGCGTGTCCTGGTCTAG-3’ |
| EC_c-template-DNA | 5’-CGAGGTCGAGCGTGTCCTGGTC-3’ |
| EC_x-template-DNA | 5’-AAGTCAAGTACTTACGCCTGGTCATTACTAGTACTGCC-3’ |
| EC-nontemplate-DNA | 5’-CGCTCGACCTCG-3’ |
| EC_a-bubble-nontemplate-DNA | 5’-CTGATCGTCGTAGCTCACACTGTCCGCTCGACCTCG-3’ |
| EC_x-tail-nontemplate-DNA | 5’-TAGTACTTGACTT-3’ |
| EC_x-bubble-nontemplate-DNA | 5’-GGCAGTACTAGTAAACTAGTATTGAAAGTACTTGACTT-3’ |
| EC_a/c-RNA | 5’-FAM-AACGGAGACCAGGAC-3’ |
| EC_b-RNA | 5’-FAM-GACCAGGAC-3’ |
| EC_x-RNA | 5’-Cy5-UAUAUGCAUAAAGACCAGGC-3’ |
